# Gallic Acid Attenuates Dimethylnitrosamine-Induced Liver Fibrosis by Alteration of Smad Phosphoisoform Signaling in Rats

**DOI:** 10.1155/2018/1682743

**Published:** 2018-12-02

**Authors:** Yuxin Chen, Ziping Zhou, Qigui Mo, Gao Zhou, Youwei Wang

**Affiliations:** ^1^Institute of TCM and Natural Products, School of Pharmaceutical Sciences, Wuhan University, Wuhan 430071, China; ^2^MOE Key Laboratory of Combinatorial Biosynthesis and Drug Discovery, Wuhan University, Wuhan 430072, China

## Abstract

Dimethylnitrosamine (DMN) is a potent hepatotoxin, carcinogen, and mutagen. In our previous study, a candidate gallic acid (GA) that widely exists in food and fruit was selected for its capability to alleviate DMN toxicity in vivo. We aimed to investigate the therapeutic potential of GA against DMN-induced liver fibrosis. During the first four weeks, DMN was administered to rats via intraperitoneal injection every other day, except the control group. GA or silymarin was given to rats by gavage once daily from the second to the sixth week. GA significantly reduced liver damage in serum parameters and improved the antioxidant capacity in liver and kidney tissues. Cytokines involved in liver fibrosis were measured at transcriptional and translational levels. These results indicate that GA exhibits robust antioxidant and antifibrosis effects and may be an effective candidate natural medicine for liver fibrosis treatment.

## 1. Introduction

Chronic liver disease is one of the major health problems, with liver cirrhosis and drug-induced liver damage being the ninth leading cause of death, in western and developing countries [1]. Liver fibrosis is a critical stage in the pathogenesis of liver damage to cirrhosis or a tumor, because liver fibrosis can be reversed, but liver cirrhosis and liver cancer cannot be reversed [2]. Therefore, conducting some drug treatments is critical to avoid deterioration during the stage of liver fibrosis. In recent years, the molecular mechanism of antifibrosis has attracted scholars, and many excellent in vivo models have been established for the study of liver fibrosis regulation. These models induced by toxins such as dimethylnitrosamine (DMN), CCl_4_, acetaminophen, or thioacetamide can represent chronic or acute/fulminant hepatitis [3]. DMN is a potent hepatotoxin, carcinogen, and mutagen. A minimal dose of 20 mg/kg DMN can cause hepatocellular necrosis and death in many species [4]. Repeated exposure to lower doses of DMN resulted in a subacute or chronic liver injury with varying degrees of necrosis, fibrosis, and nodular regeneration [5]. The DMN-induced fibrosis model is a good model to use since it has many of the similar characteristics observed in human liver fibrosis, such as an initial central hemorrhagic necrosis of the liver with rapid formation of septa and fully established micronodular cirrhosis [6]. The model also has the advantage of producing progressive and significant pathological changes, high fibrogenesis rates, and low mortality of experimental animals [7]. Therefore, our study selected DMN as the inducer of liver fibrosis.

Countries, such as China, Japan, and India, have had their own medical theories and systems, in which they become accustomed to using a variety of plants with or without current medicines in order to treat various illnesses. Recently, the beneficial effects of compounds derived from plants for the prevention and treatment of liver fibrosis have attracted extensive attention. Ramalin, an antioxidant compound derived from Antarctic lichen, has been reported to be able to prevent progression of liver fibrosis induced by DNM in rats, potentially via the Nrf2/ARE pathway [8]. Curcumin, which is a yellow pigment found in the rhizome of the spice turmeric, has shown amelioration of liver cirrhosis via its anti-inflammatory effect and suppression of HSC activity [9]. Silymarin, a clinical antifibrotic agent, is widely accepted and used for treating liver diseases. It downregulates TIMP metallopeptidase inhibitor 1 (TIMP-1) and transforming growth factor beta 1 (TGF-*β*1) expressions and suppresses collagen synthesis [10]. Silymarin is a unique flavonoid complex containing silybin, silydianin, and silychristin derived from the milk thistle plant [11]. Currently, silymarin remains the most widely clinical used antifibrotic agent in China. So we selected silymarin as the positive control in our study.

In previous study, we proved that the ethyl acetate fraction (EF) from the fruit of* Terminalia bellirica* has an antifibrotic effect in vitro [12]. Mass spectrometry analysis shows that the main components of EF are gallic acid (GA) and ellagic acid (unpublished data). GA, a type of phenolic acid with strong antioxidant effect, can be found in white, red, and black mulberry, blackberry, raspberry, strawberry, dragon fruit, guava, mangosteen, papaya, tea leaves, and other plants [13–15]. Since GA is a major component of EF, we hypothesize that GA is a major contributor to the antifibrotic effects of EF.

In this study, we aim to prove that GA has the capability to reverse liver fibrosis by using an animal model of liver fibrosis induced by DMN and to investigate biomarkers altered by GA treatment after the liver of the rat suffers subchronic injury.

## 2. Materials and Methods

### 2.1. Chemicals

All chemical reagents and commercial kits used are referred to in Supplementary Table 1.

### 2.2. Animals

A total of 48 male Sprague Dawley rats (180–200 g) were purchased from the Laboratory Animal Center of Wuhan University (Wuhan, China). They were housed in a 12 h light–dark cycle at 25 ± 2°C and at a relative humidity of 50%–70%. Animals were fed ad libitum with a standard diet of pellets and water. Animals were allowed to acclimate to housing conditions for three days prior to experimentation. This study secured clearance from the Institutional Animal Care and Use Committee (IACUC) at Center for Animal Experiment, Wuhan University, China.

### 2.3. Study Design

**Figure 1:**
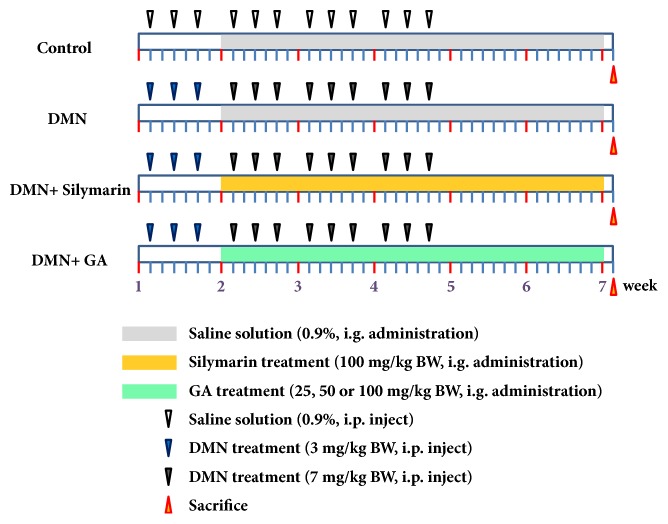
Schematic illustration for animal experiment design. DMN at 3 mg/kg BW is injected three times on the first week (blue triangle), and DMN at 7 mg/kg BW is injected three times per week from the second to the fourth week (black triangle) to all the rats except the rats in control group. Saline is injected as a control under the same regime (hollow triangle). Subsequently, GA (green column) or silymarin (yellow column) is suspended in saline and administered daily by intragastric gavage (i.g.) from the second to the sixth week. Saline is administered as a control under the same regime (gray column). Rats were weighed and sacrificed six weeks after first DMN injection (red triangle). n = 8 animals/group.

The rats were divided into six groups consisting of eight animals per group and underwent six weeks of treatment. The design of the animal work refers to Su et al. [16] and slightly modified. The schematic for DMN induction of liver fibrosis and for GA treatment refers to Figure 1. Group I was administered 0.9% saline solution as control; Group II was administered 3 mg/kg and 7 mg/kg DMN dissolved in 0.9% saline solution; Group III was the positive control group and administered silymarin, 100 mg/kg BW; Group IV was the low-dose GA group administered 25 mg/kg BW; Group V was the medium-dose GA group administered 50 mg/kg BW; and Group VI was the high-dose GA group administered 100 mg/kg BW.

### 2.4. Determination of Body and Organ Weights

The body weights were recorded before the rats were sacrificed by spine dislocation. After the rats were sacrificed, livers, kidneys, and spleens were extracted and weighed immediately.

### 2.5. Determination of Serum Biomarkers

At the end of the experiment, all rats were fasted overnight and sacrificed the next day. Blood was extracted from the heart and coagulated. The serum was obtained by centrifugation at 1000g for 10 min and stored at −20°C until use. Alanine aminotransferase (ALT), aspartate aminotransferase (AST), alkaline phosphatase (ALP), and total bilirubin (TB) were determined by the commercial kits consistent with the instructions of the manufacturers.

### 2.6. Oxidative Stress Assessment in Liver and Kidney

After the livers and kidneys were extracted and weighed, 10% liver or kidney tissue homogenate was prepared with 50 mM sodium phosphate buffer (pH 7.4) and centrifuged at 5000g for 10 min at 4°C. The supernatants were transferred into new tubes and stored at -80°C for subsequent experiments. The total protein content was determined using the Bradford method. The activities of superoxide dismutase (SOD) and catalase (CAT) and the levels of glutathione (GSH) and malondialdehyde (MDA) were determined by commercial kits, following the steps indicated in the kit manuals.

### 2.7. Hematoxylin–Eosin and Masson's Trichrome Staining

For each rat, a piece of liver tissue was fixed in 10% formalin for at least 24 h, embedded in paraffin, and cut into 5 *μ*m thick sections using a rotary microtome. The sections were stained with hematoxylin–eosin and Masson's trichrome dye, and then the slides were observed under a microscope (IX51, Olympus, Japan) to detect histopathological changes in the liver.

### 2.8. ELISA

The supernatants obtained from 10% liver homogenates were diluted in appropriate concentrations for each ELISA kit according to the range determined from the standard curve. Transforming growth factor beta 1 (TGF-*β*1), epidermal growth factor (EGF), and hydroxyproline were quantified by the corresponding assay kit consistent with the instructions of the manufacturers.

### 2.9. RNA Extraction and Real-Time Polymerase Chain Reaction

After the rats were sacrificed, the liver was quickly extracted and a small piece was ground in liquid nitrogen. After grinding in liquid nitrogen with RNase-free instrumentation, TRIzol reagent was quickly added to the ground samples. Real-time PCR was subsequently performed to detect the gene expression of alpha smooth muscle actin (*α*-SMA), platelet-derived growth factor receptor (PDGFR), TIMP-1, and tissue inhibitor of metalloproteinases 2 (TIMP-2) in triplicate using the CFX384 Touch Real-Time PCR Detection System (Bio-Rad, China). All mRNA quantification data were normalized to glyceraldehyde 3-phosphate dehydrogenase (GAPDH). For the primers, refer to Supplementary Table 2. For the amplification condition of the PCR, refer to Chen et al. [12].

### 2.10. Western Blot

The protein concentrations of the supernatants from 10% homogenate were determined by the Bradford method. Equal protein amount of supernatant from each sample was fractionated by gradient sodium dodecyl sulfate-polyacrylamide gel electrophoresis. 5% stacking gel and 12% separating gel were used for collagen I, and 5% stacking gel and 15% separating gel were used for Smad2, Smad3, p-Smad2, p-Smad3, and *β*-actin protein. The separated proteins were then transferred to polyvinylidene difluoride (PVDF) blotting membranes. Nonspecific binding was blocked by 5% nonfat milk or 5% bovine serum albumin (BSA) in TBST for 2 h and then incubated overnight at 4°C with the respective primary antibodies. The membranes were then washed and incubated with horseradish peroxidase-conjugated secondary antibodies for 1 h at room temperature. Immunoreactive bands were visualized using a chemiluminescent reagent and then exposed to X-Omat Blue XB-1 Film (Kodak, Rochester, NY) for autoradiography. The gray density of each blot was analyzed by ImageJ software (NIH, Bethesda, MD, USA).

### 2.11. Statistical Analysis

All data are shown as mean ± SEM corresponding to three replicates. IBM SPSS Statistics 20.0 was used for data analysis. Statistical differences were analyzed by one-way ANOVA, and the LSD test was performed to evaluate the significant differences of means at* p* < 0.05 level.

## 3. Results

### 3.1. Effect of GA on Rat Body and Organ Weights

Table 1 displays the body, liver, kidney, and spleen weights of the rats after 6 weeks of treatment. From the changes in body weight, we can see that the weight of rats in the DMN model group (Group II) decreased significantly (*p* <0.01) compared with the control group (Group I). The silymarin-treated (Group III) and all GA-treated groups (Groups IV– VI) had no significant effect on body weight gain compared to Group II. With increasing GA dose, the body weight gradually decreased. Hence, we hypothesize that GA has potential pharmacological effects on weight loss. The liver weight of the control group was significantly different from that of the other groups, except Group IV, and no significant difference was observed among Groups II, III, V, and VI. The statistical analysis of the kidney weights shows a similar trend with that of the liver. No statistically significant difference was observed in the spleen weights among the groups.

### 3.2. Effect of GA on Determining the Serum Biomarker

**Figure 2:**
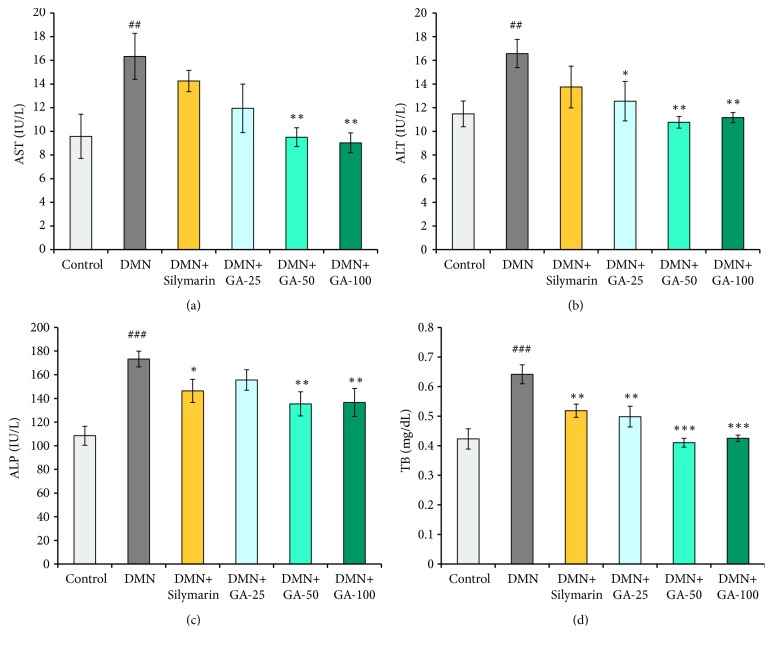
Effects of EF on the (a) AST, (b) ALT, (c) and ALP activities, and (d) the level of TB in the serum of rats with DMN-induced liver fibrosis (n = 8). The statistically significant differences are indicated by symbols (^#^*p* < 0.05, ^##^*p* < 0.01, and ^###^*p* < 0.001 compared with the control group; ^*∗*^*p* < 0.05, ^*∗∗*^*p* < 0.01, and ^*∗∗∗*^*p* < 0.001 compared with the DMN-induced group).

After treatment with GA for six weeks, serum levels of ALT, TB, ALP, and AST increased by 1.4-, 1.5-, 1.6-, and 1.7-fold, respectively, compared to control (Figures 2(b), 2(d), 2(c), and 2(a)). GA treatment with 50 or 100 mg/kg BW significantly prevented these biomarkers from elevating (*p* <0.05) compared to the DMN model group. The lowest dose of GA (25 mg/kg BW) had a comparable effect with silymarin (100 mg/kg BW) on reversing the elevation of AST and ALP levels induced by DMN (Figures 2(a) and 2(c)). For ALT and TB levels (Figures 2(b) and 2(d)), the effect of the low-dose GA was better than that of silymarin. These results show that GA has a protective effect on liver injury induced by the long-term administration of DMN in a dose-dependent manner.

### 3.3. Effect of GA on the Oxidative Stress Assessment in Liver and Kidney

**Figure 3:**
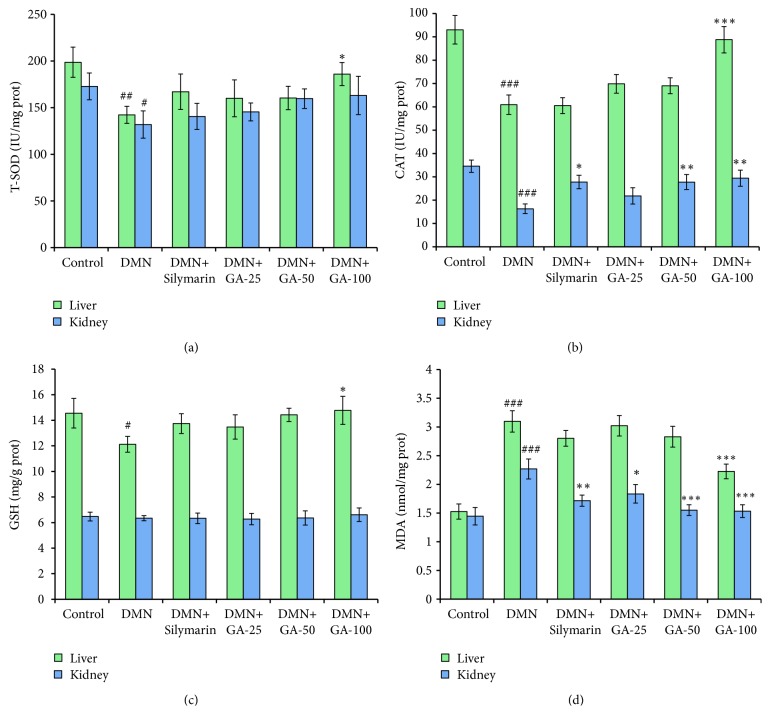
Effects of EF on the (a) SOD activity, (b) CAT activity, and the levels of (c) GSH and (d) MDA in the liver and kidney of DMN-induced liver fibrosis rats (n = 8). The statistically significant differences are indicated by symbols (^#^*p* < 0.05, ^##^*p* < 0.01, and ^###^*p *< 0.001 compared with control group; ^*∗*^*p* < 0.05, ^*∗∗*^*p* < 0.01, and ^*∗∗∗*^*p* < 0.001 compared with DMN- induced group).

In liver tissue, SOD, and CAT activities and GSH level in the DMN model groups were significantly (*p* < 0.01,* p *< 0.001,* p* < 0.05, respectively) lower than their control groups, whereas the level of MDA was significantly (*p* < 0.001) increased compared to the control group. This observation was significantly (*p* < 0.05,* p* < 0.001,* p* < 0.05, and* p* < 0.001) reversed by the high-dose GA (100 mg/kg BW) treatment (Figures 3(a)–3(d)). Although the values showed that the silymarin and low- and medium-dose GA groups affect the reversal of the changes of SOD, CAT, GSH, and MDA shown in the DMN groups, statistically significant differences were not observed. The results obtained from the four biomarkers showed that the highest dose of GA had the best effect on liver protection. Although the highest doses of GA and silymarin shared the same dose (100 mg/kg), the effect of GA was much better than the effect of silymarin.

In the kidney tissue, compared with the control groups, the DMN model significantly (*p* < 0.05 and* p* < 0.001) reduced the activities of SOD and CAT (Figures 3(a) and 3(b)), while it significantly (*p* < 0.001) elevated the MDA level (Figure 3(d)). After GA treatment (25, 50, and 100 mg/kg BW) and silymarin treatment, SOD activities were not significantly increased compared with the model group, but the values were slightly higher than that of the model group (Figure 3(a)). Compared to the model group, CAT activities increased significantly after treatment with the medium and high doses of GA and silymarin (*p* < 0.01 and* p* < 0.01) (Figure 3(b)). The three doses of GA and silymarin significantly lowered the MDA levels (*p* < 0.05,* p* < 0.001,* p* < 0.001, and* p* < 0.01, respectively) when compared to the value of model group (Figure 3(d)). Notably, the DMN model did not statistically significantly affect GSH levels (Figure 3(c)). However, the value of the model group remains the lowest, whereas the value of the highest dose GA group is closest to that of the control group. Hence, the highest dose of GA had the best protective effect on the kidney.

### 3.4. Effects of GA on the Histopathological Changes

**Figure 4:**
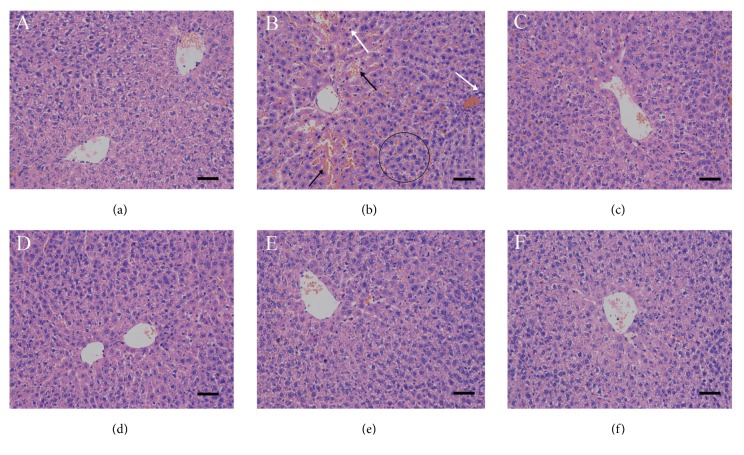
Effects of GA on the liver histological structure of rats with DMN-induced liver fibrosis (hematoxylin–eosin staining, 200×), n = 8 animals/group, scale bar: 50 *μ*m. The treatments were as follows: (a) control group, (b) DMN (3 mg/kg; 7 mg/kg), (c) DMN + silymarin (100 mg/kg), (d) DMN + GA (25 mg/kg), (e) DMN + GA (50 mg/kg), and (f) DMN + GA (100 mg/kg). Hyperplastic wire mesh-like collagen fibers around the liver cells (black circle), inflammatory cell infiltration (white arrow), and congestion in ECM (black arrow) were found in the DMN group.

Hematoxylin–eosin staining (Figure 4) of the DMN group (Figure 4(b)) showed sinus fibrosis and hyperplastic wire mesh-like collagen fibers around the liver cells (black circle). A large number of inflammatory cell infiltration (white arrow) and congestion in the extracellular matrix (black arrow) were found in the DMN group compared to control group (Figure 4(a)). GA treatment reversed these phenomena in a dose-dependent manner, which indicated that the highest dose of GA has the best liver protection effect (Figures 4(d)–4(f)).

**Figure 5:**
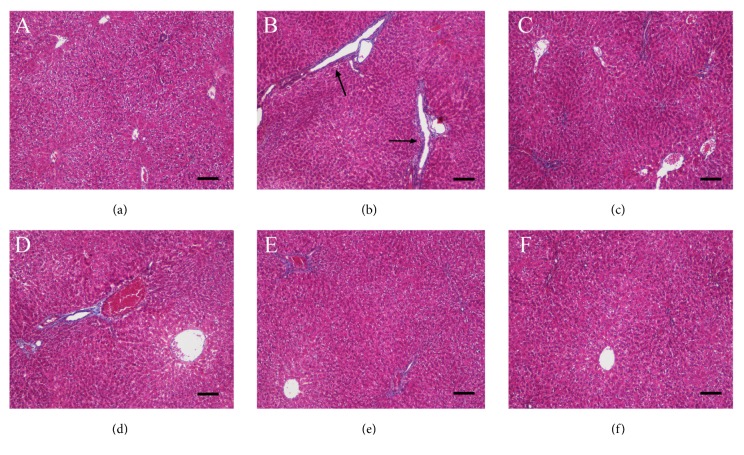
Effects of GA on the liver histological structure of rats with DMN-induced liver fibrosis (Masson staining, 100×), n = 8 animals/group, scale bar: 100 *μ*m. The treatments were as follows: (a) control group, (b) DMN (3 mg/kg; 7 mg/kg), (c) DMN + silymarin (100 mg/kg), (d) DMN + GA (25 mg/kg), (e) DMN + GA (50 mg/kg), and (f) DMN + GA (100 mg/kg). Bridging fibrosis, which formed a broad interval (black arrow) separating the liver parenchyma, was found in the DMN group.

We employed Masson trichrome staining that contains three dyes selectively to stain muscle, collagen fibers, fibrin, and erythrocytes. From Figure 5, we can see that black spots represent nuclei, red stains represent cytoplasm, muscle, erythrocytes, and blue stains represent collagen. Masson trichrome staining showed that majority of fibrous septa formed in the DMN group (Figure 5(b)). Bridging fibrosis formed a broad interval (black arrow) separating the liver parenchyma. GA treatments reversed this observation in a dose-dependent manner (Figures 5(d)–5(f)). The high dose of GA was more effective in protecting the liver from DMN damage (Figure 5(f)) compared to the low- or medium-dose GA treatment (Figures 5(d) and 5(e)). The silymarin-treated group showed constricted and reduced fibrous septa (Figure 5(c)), but the number of fibrous septa remained much more than that in the medium- or high-dose GA-treated group. Therefore, the effect of silymarin-reversing liver fibrosis was lower than the GA at the same dose.

### 3.5. Effects of GA on the Growth Factor Expression

**Figure 6:**
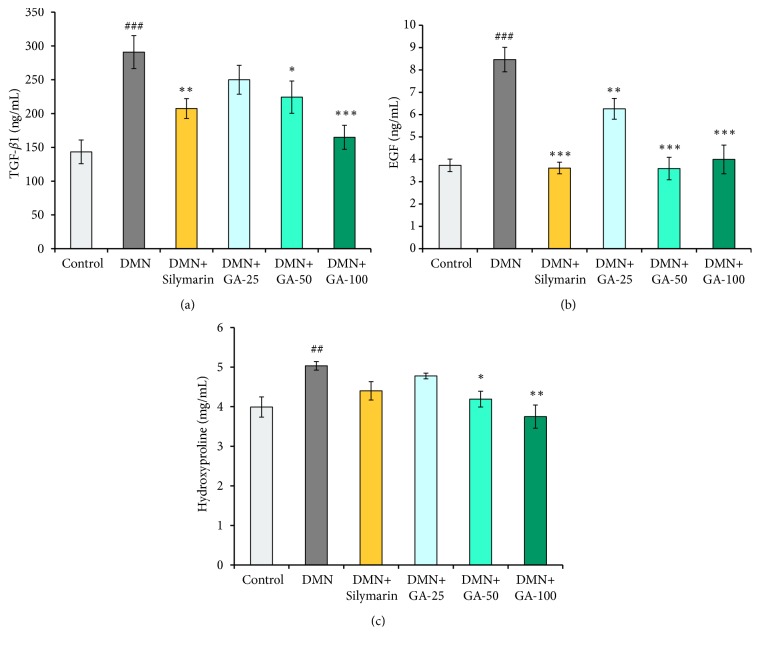
Effects of GA on the (a) TGF-*β*1, (b) EGF, and (c) hydroxyproline content in DMN-treated rat liver (n = 8). The statistically significant differences are indicated by symbols (^#^*p* < 0.05, ^##^*p* < 0.01, and ^###^*p *< 0.001 compared with the control group; ^*∗*^*p* < 0.05, ^*∗∗*^*p* < 0.01, and ^*∗∗∗*^*p* < 0.001 compared with the DMN-induced group).

Compared to the control group, DMN treatment induced a significant (*p* < 0.001) approximately 2-fold increase in TGF-*β*1 level (Figure 6(a)). After treatment with the medium/high dose of GA (50 and 100 mg/kg) or silymarin, the levels of TGF-*β*1 were significantly decreased (*p* < 0.05,* p* < 0.001, and* p* < 0.01, respectively) compared to the model group. The highest dose of GA enabled the level of TGF-*β*1 to nearly revert to the control group level. Compared with the control group, the DMN treatment significantly (*p* < 0.001) increased the content of GA (Figure 6(b)). After treatment with the three doses of GA (25, 50, and 100 mg/kg) or silymarin, the content of GA decreased significantly (*p* < 0.01,* p* < 0.001,* p* < 0.001, and* p* < 0.001, respectively) compared with the model group. The medium- and high-dose GA and silymarin treatment could significantly (*p* < 0.001) reverse the level of GA elevated by DMN to the control group level. Compared to the control group, the hydroxyproline content was significantly (*p* < 0.01) increased by the DMN modeling (Figure 6(c)), which was increased by nearly fourfold. The hydroxyproline content was decreased significantly (*p* < 0.05 and* p* < 0.01) after being treated with a medium or high dose of GA (50 and 100 mg/kg) compared to the DMN group. The high dose of GA has the best effect on reversing the abnormal increase of hydroxyproline content induced by DMN. The silymarin and low-dose GA treatment groups did not show a good inhibitory effect on hydroxyproline synthesis, which is consistent with the results shown in Figures 5(c) and 5(d). Hydroxyproline is the substrate of collagen synthesis, and fibrous septa contain large amounts of collagen; thus, hydroxyproline content is closely related to the fibrous septa formation.

### 3.6. Effects of GA on *α*-SMA, PDGFR, TIMP-1, and TIMP-2 Gene Expression

**Figure 7:**
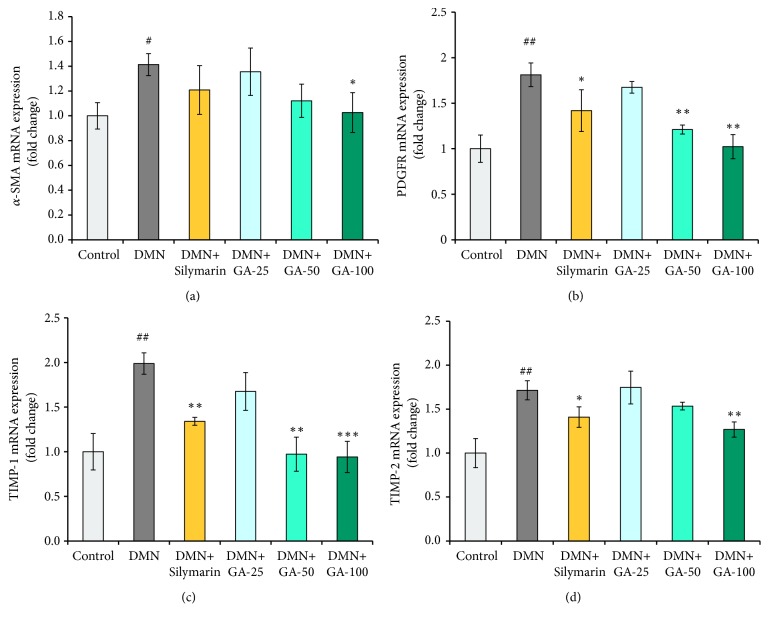
Effects of GA on the (a) *α*-SMA, (b) PDGFR, (c) TIMP-1, and (d) TIMP-2 gene expressions were measured in the DMN-treated rat liver (n = 8). The results are presented as relative changes normalized to GAPDH. The statistically significant differences are indicated by symbols (^#^*p* < 0.05, ^##^*p* < 0.01, and ^###^*p *< 0.001 compared with the control group; ^*∗*^*p* < 0.05, ^*∗∗*^*p* < 0.01, and ^*∗∗∗*^*p* < 0.001 compared with the DMN-induced group).

Compared to the control group, the DMN modeling significantly (*p* < 0.05) upregulated the mRNA expression of *α*-SMA by 1.4-fold (Figure 7(a)), and the mRNA expressions of *α*-SMA were downregulated by treating with various concentrations of GA. However, only the mRNA expression of *α*-SMA from the high-dose GA group showed a significant difference with the value obtained from the DMN model group (*p* < 0.05). Evidently, silymarin did not significantly downregulate the expression of the *α*-SMA gene, and its effect was comparable with the medium-dose GA. We then analyzed the response of the liver to GA by measuring mRNA expression of PDGFR (Figure 7(b)). A decrease in gene expression of PDGFR will reduce the response to PDGF in liver cells, further reducing the signs of liver fibrosis. The gene expression of PDGFR was significantly (*p* < 0.01) elevated by DMN treatment, but the medium- and high-dose GA or silymarin treatment played a role in reversing the PDGFR gene expression significantly (*p* < 0.01,* p* < 0.01, and* p* < 0.05, respectively). The mRNA levels of TIMP-1 and TIMP-2 (Figures 7(c) and 7(d)) were significantly (*p* < 0.01 and* p* < 0.01) higher (1.9- and 1.7-fold, respectively) in the DMN model group than those in the control group. The mRNA expression level of TIMP-1 was downregulated by GA treatment in a dose-dependent manner compared with the DMN group, but only 50 or 100 mg/kg GA treatment has a significant (*p* < 0.01 and* p* < 0.001) reversal effect on inhibiting the upregulation of TIMP-1 mRNA expression. Silymarin treatment could also significantly (*p* < 0.01) downregulate the expression of TIMP-1, but the effect was not as good as the medium- and high-dose GA treatment (Figure 7(c)). The mRNA expression levels of TIMP-2 were significantly (*p* < 0.01 and* p* < 0.05) decreased by the high-dose GA and silymarin treatment. The medium-dose GA treatment has a mild effect on reversing the elevation of the expression of TIMP-2 caused by DMN, but the mRNA expression of TIMP-2 was not nearly decreased by the low-dose GA treatment compared to the DMN group (Figure 7(d)).

### 3.7. Western Blot Analysis

**Figure 8:**
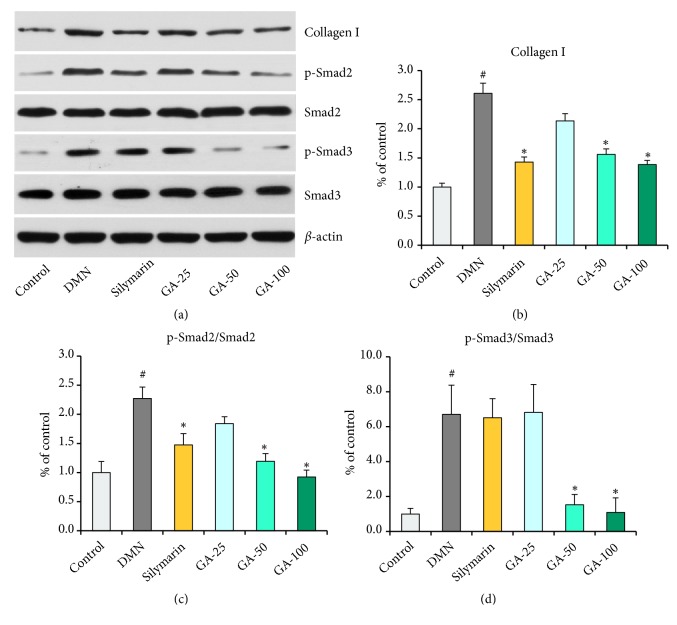
Expressions of extracellular matrix key protein and TGF-*β*/Smad signaling pathway key protein in liver. Error bars indicate the standard deviation of three independent repeats. (a) Protein expressions of collagen I, p-Smad2, Smad2, p-Smad3, and Smad3. *β*-actin as loading controls. The gray density scanning analysis of (b) Collagen I, (c) p-Smad2/Smad2, and (d) p-Smad3/Smad3 by ImageJ software. DMN, dimethylnitrosamine; GA, gallic acid. ^*∗*^*p* < 0.05, compared with the model group. #p < 0.05, compared with the control group.

Oxidative stress could alter signal transduction pathways and consequently activate key stress receptors, such as Smad2 and Smad3. The Smad protein family plays an important role in regulating cell proliferation and death in response to oxidative stress. In the present study, we analyzed the expression of Smads and p-Smads after injured liver cells were treated with silymarin and the three doses of GA (Figure 8(a)). These treatments to an extent reduced the phosphorylation-dependent activation of signaling components, such as p-Smad2 and p-Smad3 compared to that in the model group (*p *< 0.05).

The result of quantitative analysis showed that the expression of collagen I significantly (*p *< 0.05) increased in DMN group when compared to control, and silymarin and medium- and high-dose GA treatment groups significantly (*p *< 0.001) reduced the expression of collagen I (Figure 8(b)). Collagen I protein is a cell constitutive protein, continuously expressed without the induction by DMN. However, the DMN treatment induced hepatic stellate cells activation in the liver, which is the principal cellular source of collagen I protein that would be detected by Western blot [17]. Our results verified that additional collagen I was synthesized after DMN treatment (Figures 8(a) and 8(b)).

To better understand the molecular mechanism of the inhibitory effects of GA in DMN-induced liver fibrosis, we investigated the possible involvement of key TGF-*β*/Smad pathway. The results demonstrated that DMN could significantly (*p *< 0.05) activate the TGF-*β*/Smad pathway by the phosphorylation of Smad2 and Smad3 proteins. There was no significant change between the groups in terms of Smad2 or Smad3 expression, but the level of phosphorylated Smad2 or Smad3 was markedly decreased (*p *< 0.05) after treatment with silymarin and medium- and high-dose GA compared to the model group. Among the three treatments, the effect of the high-dose GA treatment group was the best, which reversed the phosphorylation level of Smad2 or Smad3 to almost normal levels (Figures 8(c) and 8(d)).

## 4. Discussion

In recent years, antifibrosis studies of plant extracts or plant-derived compounds have received increasing attention. Puerarin, naringenin, chlorogenic acid, and a variety of other compounds derived from plants have been proven to have antiliver fibrosis effect on in vivo and in vitro experiments [18–20]. GA is widely found in food and fruit, and its antifibrosis effect was investigated in the present study. The DMN-induced body weight losses of rats were clearly observed in Table 1. The body weight had a decreasing trend with treatment of increasing GA doses; thus we hypothesized that GA has a weight loss effect. This hypothesized GA function is consistent with a previous report that investigated GA as an effective and safe treatment for weight loss in an animal trial [21]. Liver and kidney weights have the corresponding change with body weight after DMN, silymarin, or GA treatments. Hence, the ratio of organ and body weight is nearly unchanged among the six groups. GA has no effect on the physiological index of the organ.

A blood test for AST is used to detect liver damage. AST is often ordered with ALT, another liver enzyme, or as part of a liver comprehensive metabolic panel to screen for and/or help diagnose liver disorders [22]. Detecting increasing enzymes levels of AST, ALT, and ALP in the serum indicates liver cell damage because large quantities of these enzymes are leaked in to the serum after liver damage. This process is usually associated with alterations in the levels of many other serum parameters, such as bilirubin, albumin, glucose, and cholesterol. The bilirubin level is an independent predictor that indicates the lack of antioxidant protection and is a possible molecular determinant for the progression of liver injury.

The primary protective mechanisms of some antifibrotic agents result from their antioxidative capabilities [23]. In this study, a series of parameters was used to measure the oxidative stress in rats. SOD is the primary enzymatic defense in the liver against the damaging effects of O^−^_2_. by converting O^−^_2_. into H_2_O_2_, which is a substrate for CAT and glutathione peroxidase (GPx) [24]. If SOD activity is low, then O^−^_2_. can interact with ·NO to form peroxynitrite (ONOO^−^), which can react to form the potent ·OH and nitrogen dioxide (NO_2_·) radicals. These radicals are highly damaging to cell proteins, lipids, and DNA [25]. We observed decreases in the activities of SOD and CAT in the DMN groups, in contrast to the control group. However, these effects were reversed by the GA treatment in a dose-dependent manner. GSH is catalyzed by GPx to reduce hydroperoxides and can effectively remove free radicals in the body to protect cells from oxidative damage. Consequently, GA enhanced the activities of antioxidant enzymes, which can lead to the increase in antioxidant capacity. The data obtained in the oxidative stress state of the liver of DMN-exposed rats show that DMN treatment leads to the accumulation of MDA as high as 2-fold of controls. Cell membranes can be modified by lipid peroxidation products, such as trans-4-hydroxy-2-nonenal, 4-hydroperoxy-2-nonenal, and MDA [26]. Lipid peroxidation products can also modulate signaling molecules and alter functions of enzymes and proteins involved in inflammation [27], which was confirmed by hematoxylin–eosin staining. We observed a large number of inflammatory cell infiltration in the DMN group. GA can help relieve the elevating trend of the level of lipid peroxidation caused by DMN. Rats treated with the higher concentration of GA had lower concentration of MDA in the liver cells.

In the kidney, we observed alarmingly low levels of SOD and CAT with DMN treatment, which can show the sensitivity of the kidney to these toxins and its inefficacy to clear reactive oxygen species (ROS).  Figure 2 shows a trend that GA at increasing doses can restore the SOD activity, but this increase was insignificant. The mechanism of GA for improving the SOD activity is unknown, but we hypothesize that GA initiates the expressions of some genes to enhance the SOD activity. The CAT activities increased significantly after being treated with various concentrations of GA compared to the DMN-treated group, which shows that GA is a good assistance for the CAT activity, possibly by enhancing the expression of the CAT gene. DMN treatment cannot significantly affect GSH levels compared to the control group. GSH is mainly synthesized in the liver and is transported to the kidney through the intestinal liver cycle. After glomerular filtration, GSH can be hydrolyzed into amino acids by the degrading enzymes located in the brush-like edge of the renal tubular cell. This process provides a reasonable explanation for the stable GSH levels we observed in the GSH content assay. In our study, various doses of GA can significantly reduce the MDA content in the kidney after DMN induction. Hence, GA is a strong reducing agent, which could reduce oxygen free radicals that can further reduce MDA formation.

The implications of this damage to an organ like the liver is extreme because this damage can hinder the capacity of the tissue to detoxify the body, leading to further exacerbated damage by the DMN toxins and leading to metabolic diseases, such as hepatic fibrosis. The increase in the number of fibrous septa by DMN treatment was confirmed by Masson trichrome staining. Collectively, these fibrous septa indicate a massive state of fibrosis occurring in response to the DMN exposure. However, this phenomenon requires at least a medium-dose GA treatment, because low-dose GA treatment did not show significant differences compared to the DMN-treated group.

The EGF family and its receptors play an important role in cell proliferation, tissue repair, and stability of normal cells. One of the main pathological features of liver fibrosis is the proliferation of fibroblasts stimulated by various growth factors. TGF-*β*1 and its receptor can stimulate fibroblast proliferation and play an important role in the pathogenesis of liver fibrosis [28]. Samarakoon et al. [29] have shown that induction of renal fibrotic genes by TGF-*β*1 requires EGF receptor (EGFR) activation, which is essential for the expression of TGF-*β*1-induced fibrotic target genes. Fuchs et al. [30] have reported that the small-molecule EGFR inhibitor, erlotinib, inhibits the activation of myofibroblastic HSCs, prevents the progression of cirrhosis, and regresses fibrosis in some animals. GA could reduce the level of EGF significantly after the animal was induced with liver fibrosis; however, whether GA is an EGFR inhibitor has yet to be determined. Hydroxyproline is an important amino acid for collagen synthesis and currently used to characterize collagens. From our study, we found that GA could significantly reduce the accumulation of hydroxyproline content in the fibrotic liver. To confirm the effects on collagen, we next measured collagen I because originally the collagen content was estimated from the hydroxyproline content of acid hydrolysates of tissue, not collagen directly [31]. Collagen I is the most abundant and ubiquitous connective tissue protein and through Western blot showed that GA could effectively reduce the deposition of collagen I in liver tissue.

The mRNA level of *α*-SMA was also markedly suppressed by the medium- and high-dose GA treatments. Hinz et al. [32] reported that accumulation of biologically active TGF-*β*1 is one of the three local events are needed to generate *α*-SMA-positive differentiated myofibroblasts. Similarly, GA significantly suppressed the mRNA level of PDGFR. Liver fibrosis is a complex dynamic process mediated by the death of hepatocytes and activation of HSCs. The generation of ROS, tumor necrosis factor-*α*, TGF-*β*, and PDGF can be implicated as a cause of hepatic fibrosis [33, 34]. The matrix metalloproteinases (MMPs) family is a major group of enzymes responsible for extracellular matrix (ECM) degradation and their activity is regulated by protein inhibitors called TIMPs [35]. Liver fibrosis, a chronic hepatic injury, is characterized by an excessive production of ECM. The balance between the activity of MMPs and the inhibitory role of TIMPs plays a key role in degrading ECM in fibrosis. After GA treatment, TIMPs had significantly altered mRNA expression compared to the DMN-treated group, suggesting that GA may be involved in the transcription of TIMP-1 and TIMP-2. The reduction in expression could lead to a reduction of their protein levels, attenuating their inhibitory effects on MMPs. Eventually, ECM degradation by MMPs was resumed to the normal activity under GA treatment.

**Figure 9:**
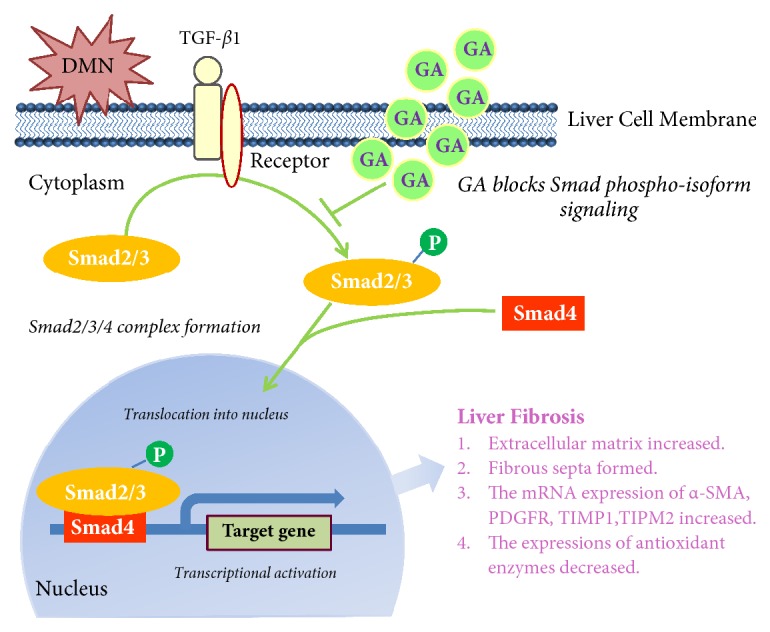
Schematic of DMN-induced liver fibrosis through activating the TGF-*β*/Smad signaling pathway and GA attenuate liver fibrosis through blocking Smad phosphorylation in liver cell.

The Smad proteins 2, 3, 4, and 7 and their roles in fibrosis have been described in some in vivo and in vitro experiments [36–38]. Angiotensin-converting enzyme (ACE) inhibitor therapy has also been shown to have an antifibrosis effect, partly because of their capability to prevent type II angiotensin-induced Smad2 nuclear translocation and inhibit transcriptional activity. Angiotensin receptor antagonists can block this important pathway and have a similar effect [39]. In response to TGF-*β*, the TGF-*β* receptor phosphorylates serine residues on the C-terminus of Smad2 and Smad3. Han et al. reported that hepcidin suppresses liver fibrosis by impeding TGF-*β*1-induced Smad3 phosphorylation in HSCs, which depends on AKT activated by a deficiency of ferroportin [40]. TGF-*β* is considered to be the major factor regulating liver carcinogenesis and accelerating liver fibrosis. Smad2 and Smad3 act as the intracellular mediators of TGF-*β* signal transduction pathway (Figure 9). Ma et al. (2014) have reported that GA is able to attenuate DMN-induced acute liver injury in mice, suggesting the potential mechanism is that increasing the expression level of hemeoxygenase-1 (HO-1) and glutathione-s-transferase alpha 3 (GSTA3) can enhance the detoxification ability of liver tissue [41]. However, in our paper, we established a subchronic toxicity model by DMN for studying liver fibrosis rather than acute liver injury and shed light on another possible mechanism of GA reversing the detrimental effect caused by DMN, involvement of key TGF-*β*/Smad pathway. In summary, our study focused on alteration of Smad phosphoisoform signaling suggesting that the Smad protein could be a potential therapeutic target for liver fibrosis.

## 5. Conclusion

The present study provides the first evidence that GA can reduce the DMN-induced liver fibrosis in rats relying on its superior antioxidant capacity and take part in the regulation of cytokines expression. Moreover, this study provides an attractive alternative strategy against liver fibrosis, because GA widely exists in plants and food.

## Figures and Tables

**Table 1:** Effects of GA on body and organ weight in rats with DMN-induced liver fibrosis (n = 8).

	Weight (g)			
Group	Body	Liver	Kidney	Spleen
Group I control	442.38± 16.72 a	15.58±0.90 a	2.85±0.15 a	1.09±0.09 a
Group II DMN	383.08±9.24 bc	12.81±0.45 b	2.44±0.09 b	0.98±0.05 a
Group III silymarin 100 (mg/kg)	368.78±12.94 b	11.66±0.74 b	2.46±0.13 b	0.99±0.07 a
Group IV GA 25 (mg/kg)	399.90±7.14 c	14.01±0.68 ab	2.64±0.10 ab	1.12±0.04 a
Group V GA 50 (mg/kg)	384.88±8.03 bc	12.66±0.45 b	2.47±0.09 b	1.11±0.06 a
Group VI GA 100 (mg/kg)	361.94±10.38 b	12.03±0.40 b	2.45±0.09 b	1.02±0.05 a

Values are Mean ± SEM., “a, b, c” means values in the same column with different letters are significantly different from each other (*p*<0.05).

## Data Availability

The data used to support the findings of this study are available from the corresponding author upon request.

## Supplementary Materials

Supplementary Materials Table S1. Reagents and Chemicals Manufacturer List. Table S2. Real-Time PCR Primers for Analysis.
