# Supplementary material for: Gallic Acid Attenuates Dimethylnitrosamine-Induced Liver Fibrosis by Alteration of Smad Phosphoisoform Signaling in Rats
Source: Biomed Res Int. 2018 Dec 2;2018:1682743. doi: 10.1155/2018/1682743 (PMC6304566; doi:10.1155/2018/1682743)
Supplement: Supplementary Materials — Table S1. Reagents and Chemicals Manufacturer List. Table S2. Real-Time PCR Primers for Analysis. [file 1682743.f1.docx]

Table S1. Reagents and Chemicals Manufacturer List

| **Chemicals or Reagents Name** | **Manufacturer** |
| --- | --- |
| Gallic acid (purity>98%) | Sangon Biotech Co., Ltd. (Shanghai, China) |
| Dimethylnitrosamine (purity>99%) | Sigma (Sigma-Aldrich, USA) |
| Alanine aminotransferase (ALT) kit | Institute of Biological Engineering of Nanjing Jiancheng (Nanjing, China) |
| Aspartate aminotransferase (AST) kit | Institute of Biological Engineering of Nanjing Jiancheng (Nanjing, China) |
| Alkaline phosphatase (ALP) kit | Institute of Biological Engineering of Nanjing Jiancheng (Nanjing, China) |
| Total bilirubin (TB) kit | Institute of Biological Engineering of Nanjing Jiancheng (Nanjing, China) |
| Superoxide dismutase (SOD) kit | Institute of Biological Engineering of Nanjing Jiancheng (Nanjing, China) |
| Catalase (CAT) kit | Institute of Biological Engineering of Nanjing Jiancheng (Nanjing, China) |
| Glutathione (GSH) kit | Institute of Biological Engineering of Nanjing Jiancheng (Nanjing, China) |
| Malondialdehyde (MDA) kit | Institute of Biological Engineering of Nanjing Jiancheng (Nanjing, China) |
| TGF-β1 ELISA kit | NeoBioscience Technology (Shanghai, China) |
| EGF ELISA kit | NeoBioscience Technology (Shanghai, China) |
| Hydroxyproline ELISA kit | Shanghai Bogoo Biological Technology Co., Ltd. (Shanghai, China) |
| Primer synthesis | Sangon Biotech (Shanghai, China) |
| PrimeScript RT reagent kit | TaKaRa Biotechnology Co. (Dalian, China) |
| dNTP | TaKaRa Biotechnology Co. (Dalian, China) |
| Taq DNA polymerase | TaKaRa Biotechnology Co. (Dalian, China) |
| iQ™ SYBR Green supermix | Bio-Rad (Shanghai, China) |
| 5 × loading buffer | Beyotime Biotechnology Co., Ltd. (Shanghai, China) |
| 30% acrylamide | Beyotime Biotechnology Co., Ltd. (Shanghai, China) |
| β-actin monoclonal antibody | Proteintech Group, Inc. (USA) |
| Collagen I rabbit source antibody | Rockland Immunochemicals Inc. (USA) |
| p-Smad2 rabbit source antibody | Cell Signaling Technology, Inc. (USA) |
| Smad2 rabbit source antibody | Cell Signaling Technology, Inc. (USA) |
| p-Smad3 rabbit source antibody | Cell Signaling Technology, Inc. (USA) |
| Smad3 rabbit source antibody | Cell Signaling Technology, Inc. (USA) |
| Goat anti rabbit secondary antibody | Beijing Ray Antibody Biotech (Beijing, China) |

Table S2. Real-time PCR Primers for Analysis

| Gene | Direction | Sequence |
| --- | --- | --- |
| GAPDH | Forward | GGCATCGTGGAAGGGCTCAT |
|  | Reverse | AGGGATGATGTTCTGGGCTGC |
| α-SMA | Forward | TTCGTTACTACTGCTGAGCGTGAGA |
|  | Reverse | AAAGATGGCTGGAAGAGGGTC |
| PDGFR | Forward | CATTGGGGACAGGGAAGTGGAC |
|  | Reverse | CCTGATGGTGATGCTCTCGC |
| TIMP1 | Forward | GCCTACACCCCAGCCAT |
|  | Reverse | ATGCCAGGGAACCAGGAAGC |
| TIMP2 | Forward | GGCAACCCCATCAAGAGGATTCAAT |
|  | Reverse | CACACTGCTGAGGAGGGG |
